# Association between the triglyceride-glucose index and hyperuricemia in an apparently healthy population with zero metabolic syndrome components: a cross-sectional study

**DOI:** 10.3389/fendo.2026.1811350

**Published:** 2026-04-17

**Authors:** Xing Guan, Yu-Qiang Zuo, Zhi-Hong Gao, Ping-Yong Feng, Xue-Kun Zhang, Cong Sun, Li-Jun Wang

**Affiliations:** 1 Department of Physical Examination Center, People’s Hospital of Shijiazhuang City, Shijiazhuang, Hebei, China; 2 Department of Physical Examination Center, The Second Hospital of Hebei Medical University, Shijiazhuang, Hebei, China; 3 Department of Medical Imaging Center, The Second Hospital of Hebei Medical University, Shijiazhuang, Hebei, China; 4 Department of Otorhinolaryngology, People’s Hospital of Shijiazhuang City, Shijiazhuang, Hebei, China

**Keywords:** dose-response relationships, effect modifications, hyperuricemia, insulin resistance, triglyceride-glucose index

## Abstract

**Objective:**

To investigate the association between the triglyceride-glucose (TyG) index and hyperuricemia (HUA) in an apparently healthy population, specifically defined as individuals presenting with zero components of metabolic syndrome (MetS) components. Unlike previous studies on general populations, this study focuses on a “metabolically clean” cohort to explore the early predictive value of the TyG index.

**Methods:**

This cross-sectional study included 1,181 metabolically healthy participants who did not meet even a single criterion for MetS (median age of 38.00 years; interquartile range: 33.00 - 47.00 years). Participants were stratified by TyG index quartiles (Q1-Q4). Multivariable logistic regression models were constructed to assess the association between the TyG index (as both a continuous and categorical variable) and HUA. Subgroup analyses were conducted to evaluate the robustness of this association across various clinical strata, and restricted cubic splines (RCS) were employed to characterize dose-response relationships.

**Results:**

The prevalence of HUA increased significantly across TyG quartiles (Q1: 4.01%; Q4: 11.50%, *P* for trend <0.001). In the fully-adjusted model (correcting for sex, age, body mass index, smoking status, drinking status, low-density lipoprotein cholesterol and estimated glomerular filtration rate), each unit increase in the TyG index was associated with a 2.208-fold increased risk of HUA [odds ratio (OR) =2.208; 95% confidence interval (CI): 1.088–4.481, *P* = 0.021]. Compared to Q1, participants in Q4 exhibited a significantly higher risk (OR = 1.916; 95% CI: 0.908–4.044, *P* = 0.088). Importantly, RCS analysis revealed a linear dose-response relationship between the TyG index and HUA risk (*P* for overall association =0.053, *P* for non-linearity =0.217). Receiver operating characteristic analysis demonstrated a modest discriminatory ability of the TyG index (area under curve=0.631) for HUA prediction.

**Conclusion:**

In an apparently healthy population with zero MetS components, the TyG index is independently and positively associated with HUA in linear dose-response manner. Our findings highlight the potential of the TyG index as a tool for risk stratification in primary prevention settings, particularly for ruling out HUA, even in individuals without overt metabolic disorders.

## 1 Introduction

Hyperuricemia (HUA), defined by elevated serum uric acid levels, has emerged as a pivotal public health concern due to its established pathological links with gout, chronic kidney disease, and adverse cardiometabolic outcomes (1, 2). Beyond these chronic conditions, recent evidence from acute-care settings has further underscored the broader clinical significance of serum uric acid (SUA) abnormalities. Studies in real-world emergency populations have demonstrated that both hyperuricemia and hypouricemia are potent predictors of adverse clinical outcomes, including an increased risk of stroke and higher long-term mortality (3, 4). These findings from acute clinical settings highlight the critical necessity of identifying early indicators of SUA dysregulation, such as the triglyceride-glucose (TyG) index, even in asymptomatic and metabolically healthy individuals.

Although HUA has been conventionally linked to dietary factors and renal excretory determinants, growing evidence underscores the fundamental role of insulin resistance (IR) in its pathogenesis (5–7). The triglyceride-glucose (TyG) index has subsequently been validated as a simple, reliable, and cost-effective surrogate marker of IR, showing a strong correlation with the gold-standard hyperinsulinemic-euglycemic clamp results across various populations (8).

In recent years, several epidemiological studies have explored the association between the TyG index and HUA in general or clinical populations, such as patients with type 2 diabetes or hypertension (9–11). These findings collectively suggest a potential role of IR, as reflected by the TyG index, in promoting HUA, possibly through mechanisms involving impaired renal urate excretion and increased systemic inflammation (12, 13). However, despite these insights, significant knowledge gaps persist. First, most prior investigations have focused on populations already exhibiting overt metabolic dysfunction, leaving it unclear whether the TyG-HUA association persists in apparently healthy individuals free from any individual components of metabolic syndrome (MetS)-a population in whom early metabolic risk stratification is paramount for primary prevention. Furthermore, while sexual dimorphism in both IR (14) and uric acid metabolism (15) is well-documented, it remained to be determined whether the TyG-HUA association is consistent across sexes within this unique, zero-component cohort. Clarifying whether this relationship is independent of sex is critical for ensuring the generalizability of the TyG index as a stable marker for personalized risk assessment.

To address these gaps, we conducted a cross-sectional study in an apparently healthy adult population strictly excluding established MetS. We aimed to: (1) evaluate the independent association between the TyG index (both as a continuous variable and in quartiles) and the prevalence of HUA, (2) characterize the dose-response relationship between the TyG index and HUA risk using restricted cubic spine (RCS); (3) assess the predictive performance of the TyG index for HUA. Our findings aim to provide novel insights into the role of early IR, quantified by a readily available biomarker, in HUA development among metabolically resilient individuals, thereby informing potential risk stratification approaches in clinical practice.

## 2 Methods

### 2.1 Study design and population

This retrospective cross-sectional study was conducted using data from adults (aged ≥18 years) who underwent routine health screening at the Physical Examination Center of the Second Hospital of Hebei Medical University between January 2024 and December 2024. Notably, the participants were recruited from a universal annual health screening program provided as an employer welfare benefit. This ensures that the study population is not a “convenience sample” of individuals who voluntarily sought checkups due to specific health concerns (which would indeed introduce healthy volunteer bias). Instead, it captures a representative cross-section of the working population who undergo screening as a routine institutional process. This retrospective study was approved by the Research Ethics Committee of the Second Hospital of Hebei Medical University (Approval no. 2022-R341) and conducted in accordance with the Declaration of Helsinki. Since the study involved the analysis of de-identified, retrospective clinical data collected during routine health screenings, the requirement for informed consent was waived by the Research Ethics Committee. All data were analyzed anonymously to ensure participant confidentiality.

### 2.2 Inclusion and exclusion process

**Figure 1:**
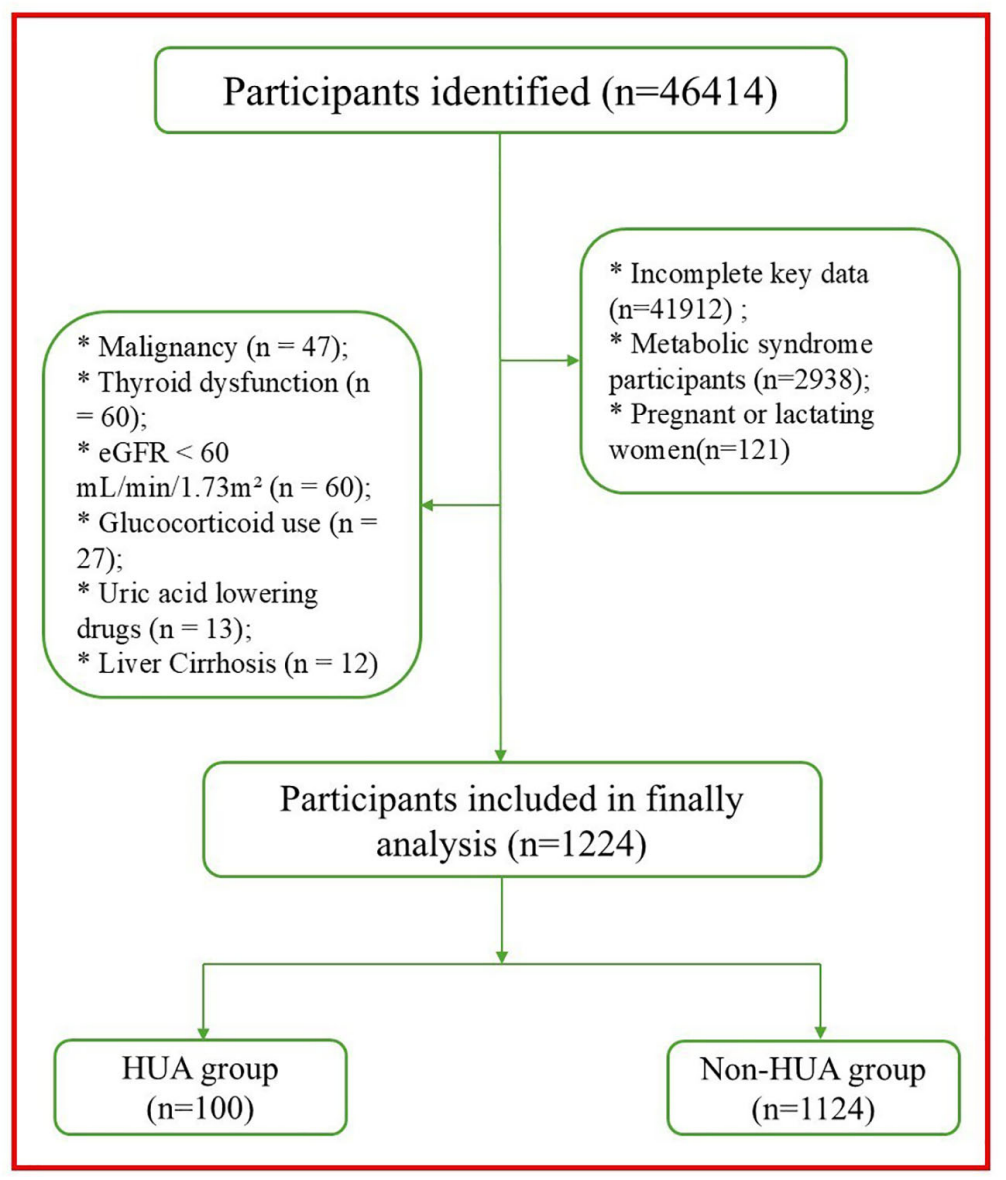
Participants enrollment flowchart.

To ensure the reliability of the baseline metabolic profile, we applied stringent inclusion and exclusion criteria. Initially, participants were included if they were aged ≥18 years and possessed complete baseline records, including demographic data, anthropometric measurements, and laboratory panels (renal and hepatic function markers, lipid profiles, and fasting glucose). We aimed to define a strictly “metabolically healthy” study cohort by excluding potential confounders. The exclusion criteria were hierarchically applied as follows: (1) MetS Abnormality Exclusion: To ensure a strictly “metabolically clean” cohort, we excluded any participants who met one or more of the five diagnostic components for MetS according to the 2024 Chinese Guideline (16)[specifically: abdominal obesity, hyperglycemia, hypertension, hypertriglyceridemia, or low high-density lipoprotein cholesterol (HDL-C)[. Consequently, the final study population consisted exclusively of individuals with zero MetS components. By excluding those with overt MetS while retaining individuals with high-normal or borderline metabolic markers (such as pre-diabetic glucose levels), we aimed to evaluate the TyG index’s utility in a population where early intervention is most impactful. (2) Medication and Comorbidity Interference: To eliminate pharmacological or pathological interference with uric acid metabolism, we excluded participants who: (a) were currently using uric acid-lowering agents, diuretics (thiazides or loop diuretics), or glucocorticoids; (b) had significant renal impairment, defined as an estimated glomerular filtration rate (eGFR) < 60 mL/min/1.73 m² (17); (c) had a history of gout, malignancy, or liver cirrhosis; or (d) exhibited thyroid dysfunction (defined as thyroid stimulating hormone levels outside the reference range of 0.5–5.0 mIU/L or current use of thyroid-related medications). (e) with ultrasonographic evidence of fatty liver (non-alcoholic fatty liver disease, NAFLD). (3) Physiological Status: Women who were pregnant or lactating were excluded. The detailed selection flowchart is presented in **Figure 1**.

### 2.3 Data collection and clinical measurements

Anthropometric and clinical data were collected by trained medical personnel following standard operating procedures. Participants underwent height and weight measurements wearing light clothing and no shoes. Body mass index (BMI) was calculated as weight (kg) divided by height squared (m²). Systolic blood pressure (SBP) and diastolic blood pressure (DBP) were measured using an automated sphygmomanometer (Omron HEM-7136) after the participant had rested in a seated position for at least 5 minutes. Venous blood samples were obtained following an overnight fast of 8–12 hours. Serum samples were separated by centrifugation (3000 rpm for 10 min) and analyzed using an automated biochemical analyzer (Beckman Coulter AU5800). Laboratory parameters included fasting plasma glucose (FPG), total cholesterol (TC), triglycerides (TG), high-density lipoprotein cholesterol (HDL-C), low-density lipoprotein cholesterol (LDL-C), urea, serum uric acid (SUA), and serum creatinine (Scr). The eGFR was calculated using the Chronic Kidney Disease Epidemiology Collaboration (CKD-EPI) equation (17). The TyG index was calculated as: TyG = Ln [TG (mg/dL) × FPG (mg/dL)/2] (18). Although TG and FPG were reported in mmol/L in the descriptive tables to adhere to international units, they were converted to mg/dL before TyG computation using the following conversion factors: 1 mmol/L = 18 mg/dL for FPG and 1 mmol/L = 88.5 mg/dL for TG. We have verified that all TyG values were derived using these converted units to ensure accuracy.

### 2.4 Variable definitions

MetS: According to the 2024 Chinese Guideline (16), MetS was defined by the presence of three or more of the following: (1) abdominal obesity (waist circumference ≥90 cm for men, ≥85 cm for women); (2) hyperglycemia (FPG ≥6.1 mmol/L or previously diagnosed diabetes); (3) hypertension (SBP/DBP ≥130/85 mmHg or treated hypertension); (4) hypertriglyceridemia (TG ≥1.70 mmol/L); and (5) low HDL-C (<1.04 mmol/L). In this study, these criteria were employed as exclusion criteria: only individuals who fulfilled none (zero) of these five criteria were eligible for inclusion. Hyperuricemia (HUA): HUA was defined as SUA levels >420 μmol/L (7.0 mg/dL) in men and >360 μmol/L (6.0 mg/dL) in women (19). NAFLD: Participants were diagnosed according to the 2018 American Association for the Study of Liver Diseases guidelines (20). The diagnosis was established based on the following criteria: (1) Presence of hepatic steatosis detected by abdominal ultrasonography; (2) Absence of significant alcohol consumption (defined as <30 g/day for men and <20 g/day for women); and (3) Exclusion of secondary causes of hepatic steatosis, such as viral hepatitis, autoimmune liver disease, or use of steatogenic medications.

### 2.5 Statistical analysis

Data analysis was performed using R software (version 4.4.3; R Foundation for Statistical Computing, Vienna, Austria). Continuous variables were assessed for normality via the Kolmogorov–Smirnov test and visual inspection of histograms. Normally distributed variables were expressed as mean ± standard deviation (SD) and compared using the independent Student’s t-test, while non-normally distributed data were presented as medians (interquartile range, IQR) and compared using the Mann–Whitney U test. Categorical variables were expressed as frequencies (percentages) and compared using the Chi-square test.

To evaluate the association between the TyG index and HUA, multivariable logistic regression models were employed in a stepwise manner: Model 1 was unadjusted; Model 2 was adjusted for age and sex; Model 3 was further adjusted for BMI, eGFR, LDL-C, smoking, and drinking status. Linear trends across TyG index quartiles were assessed by treating the quartiles as an ordinal variable (assigned values 1–4) in both linear and logistic regression models. For non-normally distributed continuous variables, the Jonckheere-Terpstra test was utilized to ensure robust trend estimation.

To explore potential non-linearity in the association between the TyG index and HUA risk, RCS analysis was performed with four knots (at the 5th, 35th, 65th, and 95th percentiles), with non-linearity assessed using the likelihood ratio test. Subgroup analyses were conducted based on age strata, sex, smoking, and drinking status, with *P*-values for interaction calculated to evaluate the consistency of the findings. The predictive performance of the TyG index was evaluated using Receiver Operating Characteristic (ROC) curve analysis. To assess the stability of the ROC results, internal validation was performed using a bootstrap method with 1,000 iterations to calculate the optimism-corrected area under curve (AUC) and its 95% confidence interval (CI). All tests were two-tailed, and a P-value < 0.05 was considered statistically significant.

## 3 Results

### 3.1 Baseline characteristics of the study population

A total of 1,181 apparently healthy participants were included; the median age of the overall study population was 38.00 years (IQR: 33.00 - 47.00 years). Among the participants, 174 (14.73%) were male and 1007 (85.27%) were female. The study participants were stratified into quartiles based on their TyG index: Q1 (lowest), Q2, Q3, Q4 (highest). As shown in **Table 1**, significant differences across TyG quartiles were observed for age, sex, BMI, blood pressure, lipid profiles, glucose, renal function (all *P* < 0.001) except for urea (*P* = 0.221), pulse (*P* = 0.075) and smoking status (*P* = 0.061). Notably, there was a progressive increase in the prevalence of HUA from Q1 to Q4 (4.01% to 11.50%, *P* for trend <0.001).

**Table 1:** Baseline characteristics of the study population (stratified by TyG index quartiles).

Variables	Total (n = 1181)	Q1 (n = 299)	Q2 (n = 297)	Q3 (n = 298)	Q4 (n = 287)	*P*	*P* for trend
Age (Years)	38.00 (33.00, 47.00)	34.00 (31.00,39.50)	37.00 (33.00,45.00)	41.00 (34.00,49.00)	42.00 (35.00,51.50)	<0.001	<0.001
BMI (kg/m^2^)	21.51 (20.19, 22.98)	21.08 (19.69,22.23)	21.09 (19.61,22.73)	21.66 (20.37,23.04)	22.35 (20.80,23.73)	<0.001	<0.001
Pulse	79.00 (72.00, 87.00)	77.00 (71.00,85.50)	80.00 (72.00,88.00)	79.00 (71.25,87.75)	80.00 (72.50,86.00)	0.075	0.067
SBP (mmHg)	113.00 (105.00, 120.00)	111.00 (104.00,118.00)	111.00 (104.00,119.00)	113.00 (105.00,120.00)	116.00 (109.00,122.00)	<0.001	<0.001
DBP (mmHg)	68.00 (63.00, 74.00)	67.00 (61.00,72.00)	68.00 (62.00,74.00)	69.00 (64.00,73.00)	70.00 (65.00,76.00)	<0.001	<0.001
WC (cm)	77.00 (72.00, 80.00)	75.00 (70.00,79.00)	75.00 (70.00,80.00)	77.00 (73.00,81.00)	79.00 (74.50,83.00)	<0.001	<0.001
HC (cm)	95.00 (92.00, 98.00)	94.00 (90.00,98.00)	95.00 (91.00,98.00)	95.00 (92.00,98.00)	96.00 (93.00,99.00)	<0.001	<0.001
TG (mmol/L)	0.84 (0.65, 1.08)	0.55 (0.51,0.60)	0.74 (0.69,0.80)	0.96 (0.88,1.03)	1.29 (1.16,1.44)	<0.001	<0.001
LDL (mmol/L)	2.69 (2.27, 3.20)	2.39 (2.04,2.81)	2.60 (2.20,3.04)	2.81 (2.39,3.32)	3.07 (2.58,3.60)	<0.001	<0.001
TC (mmol/L)	4.64 (4.16, 5.22)	4.29 (3.91,4.76)	4.51 (4.07,5.03)	4.69 (4.28,5.32)	5.04 (4.52,5.63)	<0.001	<0.001
HDL (mmol/L)	1.56 (1.37, 1.74)	1.61 (1.44,1.83)	1.56 (1.39,1.80)	1.52 (1.31,1.71)	1.51 (1.32,1.68)	<0.001	<0.001
FPG (mmol/L)	4.83 (4.55, 5.11)	4.70 (4.45,4.95)	4.80 (4.52,5.08)	4.84 (4.58,5.12)	5.00 (4.75,5.32)	<0.001	<0.001
Urea (mmol/L)	4.23 (3.59, 5.03)	4.28 (3.66,5.01)	4.17 (3.49,4.89)	4.16 (3.56,5.05)	4.37 (3.68,5.22)	0.221	0.344
Scr (mmol/L)	63.60 (57.40, 71.60)	60.60 (55.65,66.95)	63.50 (57.60,70.40)	65.50 (59.18,72.40)	66.30 (59.25,75.55)	<0.001	<0.001
SUA (mmol/L)	269.00 (233.00, 315.00)	253.00 (222.50,291.00)	271.00 (231.00,308.00)	273.00 (235.00,322.75)	288.00 (248.50,342.00)	<0.001	<0.001
TyG	8.08 (7.81, 8.35)	7.65 (7.54,7.73)	7.95 (7.89,8.03)	8.21 (8.15,8.28)	8.53 (8.44,8.64)	<0.001	<0.001
eGFR	105.47 (94.16, 115.33)	113.36 (101.90,119.56)	105.26 (94.91,114.88)	101.94 (92.54,112.78)	100.66 (90.62,111.71)	<0.001	<0.001
Sex, n (%)						<0.001	<0.001
Female	1007 (85.27)	274 (91.64)	266 (89.56)	247 (82.89)	220 (76.66)		
Male	174 (14.73)	25 (8.36)	31 (10.44)	51 (17.11)	67 (23.34)		
Smoke, n (%)						0.061	0.008
No	1135 (96.10)	294 (98.33)	287 (96.63)	283 (94.97)	271 (94.43)		
Yes	46 (3.90)	5 (1.67)	10 (3.37)	15 (5.03)	16 (5.57)		
Drink, n (%)						<0.001	<0.001
No	1078 (91.28)	284 (94.98)	281 (94.61)	267 (89.60)	246 (85.71)		
Yes	103 (8.72)	15 (5.02)	16 (5.39)	31 (10.40)	41 (14.29)		
HUA, n (%)						0.002	<0.001
No	1089 (92.21)	287 (95.99)	280 (94.28)	268 (89.93)	254 (88.50)		
Yes	92 (7.79)	12 (4.01)	17 (5.72)	30 (10.07)	33 (11.50)		

BMI, body mass index; DBP, diastolic blood pressure; FPG, fasting plasma glucose; HDL-C, high-density lipoprotein cholesterol; HUA, hyperuricemia; LDL-C, low-density lipoprotein cholesterol; SBP, systolic blood pressure; Scr, serum creatinine; SUA, serum uric acid; TC, total cholesterol; TG, triglycerides; TyG, triglyceride-glucose index; WC, waist circumference.

Data are presented as median (interquartile range) for continuous variables or number (percentage) for categorical variables.

The P value for trend across TyG index quartiles was derived from linear regression analysis for continuous variables and the Cochran-Armitage trend test for categorical variables.

### 3.2 Association between the TyG index and HUA

#### 3.2.1 TyG index as a continuous variable

In both crude and adjusted models, the TyG index—analyzed as a continuous variable—exhibited a significant positive association with HUA. After stringent adjustment for sex, age, BMI, eGFR, LDL-C, smoking status, and drinking status, each unit increase in the TyG index corresponded to an OR of 2.208 (95% CI: 1.088–4.481, *P* = 0.028) **Table 2**.

**Table 2:** Multivariable-adjusted odds ratios for the association between the TyG index (continuous) and hyperuricemia (HUA).

Model	B	OR (95% CI)	*P*
Model 1	1.271	3.566 (1.914-6.643)	<0.001
Model 2	1.198	3.313 (1.724-6.367)	<0.001
Model 3	0.792	2.208 (1.088-4.481)	0.028

Model 1, Crude; Model 2, adjusted by Sex, Age; Model 3, Adjusted by Sex, Age, BMI, eGFR, LDL-C, Smoking, Drinking.

#### 3.2.2 TyG Index as a categorical variable (quartiles)

When analyzed by quartiles, the risk of HUA increased across the gradient of the TyG index. After adjusting for age, sex, BMI, smoking, drinking, LDL, and eGFR (Model 3), a significant positive trend was observed between TyG index quartiles and the risk of HUA (*P* for trend = 0.041). Although the comparison between the highest and lowest quartiles did not reach statistical significance (OR = 1.916, 95% CI: 0.908–4.044, *P* = 0.088), the dose-response relationship remained evident **Table 3**.

**Table 3:** Multivariable-adjusted odds ratios for the association between TyG index quartiles and hyperuricemia (HUA).

Model	Q1 (ref)	Q2		Q3		Q4		*P* for trend
		OR (95% CI)	*P*	OR (95% CI)	*P*	OR (95% CI)	*P*	
Model 1	1.00	1.452 (0.681-3.096)	0.334	2.677 (1.343-5.337)	0.005	3.107 (1.517-6.145)	<0.001	<0.001
Model 2	1.00	1.516 (0.707-3.250)	0.285	2.717 (1.342-5.502)	0.005	2.988 (1.474-6.056)	0.002	<0.001
Model 3	1.00	1.218(0.560-2.648)	0.619	2.100 (1.022-4.316)	0.044	1.916 (0.908-4.044)	0.088	0.041

Model 1, Crude; Model 2, adjusted by Sex, Age; Model 3, Adjusted by Sex, Age, BMI, eGFR, LDL-C, Smoking, Drinking; Model 4, Adjusted by Sex, Age, BMI, eGFR, LDL-C, Smoking, Drinking and NAFLD.

Q, TyG Quartiles.

### 3.3 Predictive performance and further analyses

#### 3.3.1 Predictive value of the TyG index for HUA

**Figure 2:**
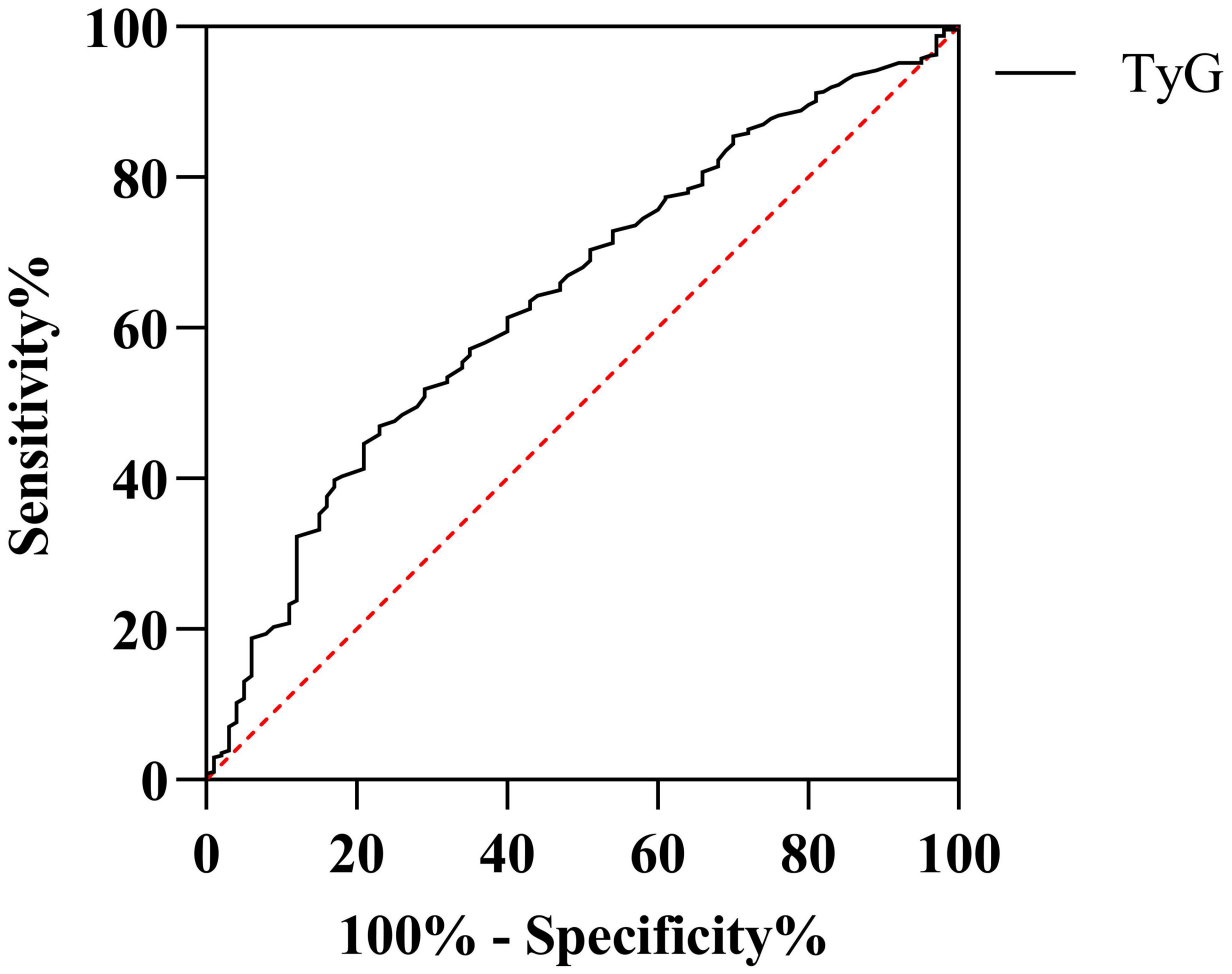
ROC curves for predicting HUA using the TyG index.

The discriminative ability of the TyG index for predicting HUA was evaluated using ROC curve analysis (**Figure 2**). The TyG index demonstrated a modest but statistically significant area under the curve (AUC) of 0.631 (95% CI: 0.603–0.659). To ensure the stability of this performance estimate, internal validation via 1,000 bootstrap iterations was performed, yielding a consistent mean AUC of 0.631 (95% CI: 0.570–0.687). The optimal cut-off value, determined by the maximum Youden index, was 8.05. At this threshold, the TyG index exhibited a sensitivity of 75.0% and a specificity of 48.1%. Despite a relatively low positive predictive value (PPV) of 10.88%, the diagnostic performance yielded a robust negative predictive value (NPV) of 95.80%. Given the low prevalence of HUA (7.79%) in this strictly metabolically healthy population, the high NPV suggests that the TyG index may serve as a potential tool for risk stratification in primary prevention settings, particularly for ruling out HUA.

#### 3.3.2 Subgroup analyses

**Figure 3:**
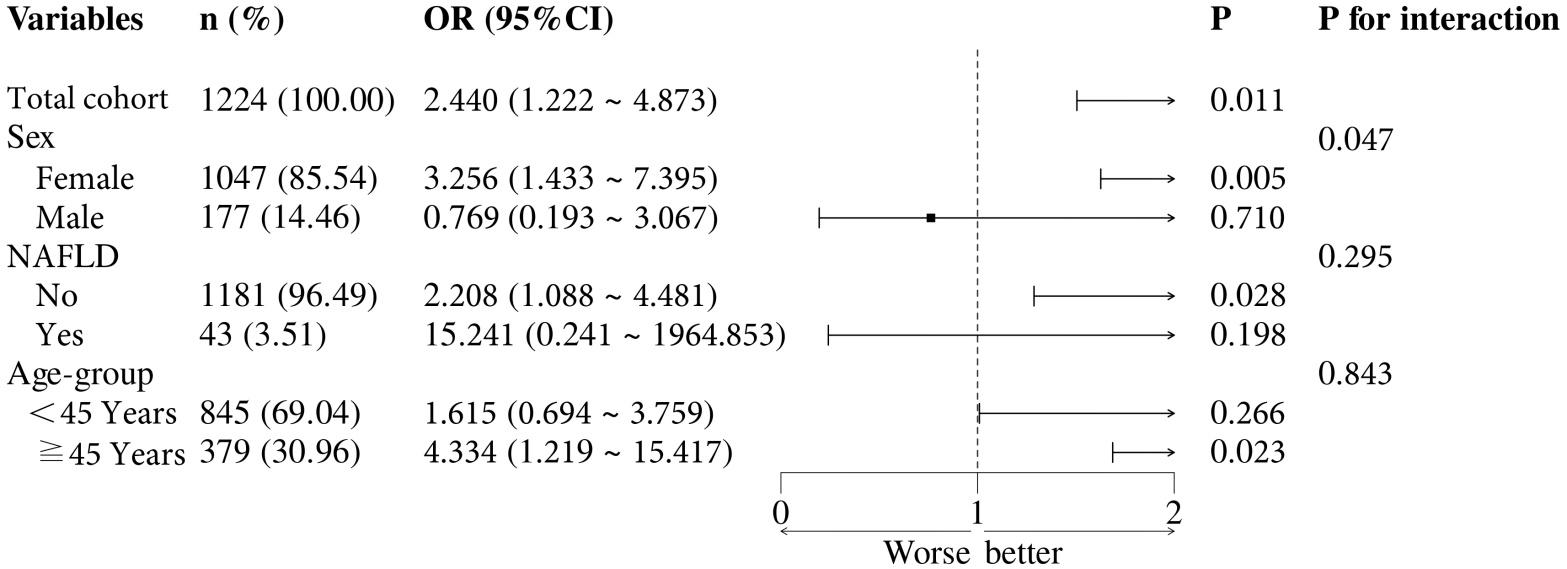
Forest plot of subgroup analyses for the association between the TyG index and HUA.

To further evaluate the robustness of the association between the TyG index and the risk of HUA, we performed stratified analyses across various clinical subgroups, including sex, age (<45 years, ≧ 45 years), smoking status and drinking status (**Figure 3**). For these analyses, the TyG index was dichotomized into high and low groups based on the median value within the study population. The positive association between a higher TyG index and an increased risk of HUA remained highly consistent across all evaluated strata. Notably, no significant effect modifications were observed for sex (*P* for interaction = 0.272), age (*P* for interaction = 0.225), smoking status (*P* for interaction = 0.134), or drinking status (*P* for interaction = 0.395). These results indicate that the predictive value of the TyG index for HUA is independent of these demographic and lifestyle factors within this metabolically clean cohort, further reinforcing its potential utility as a stable marker for risk stratification.

#### 3.3.3 Dose-response relationship via RCS analysis

**Figure 4:**
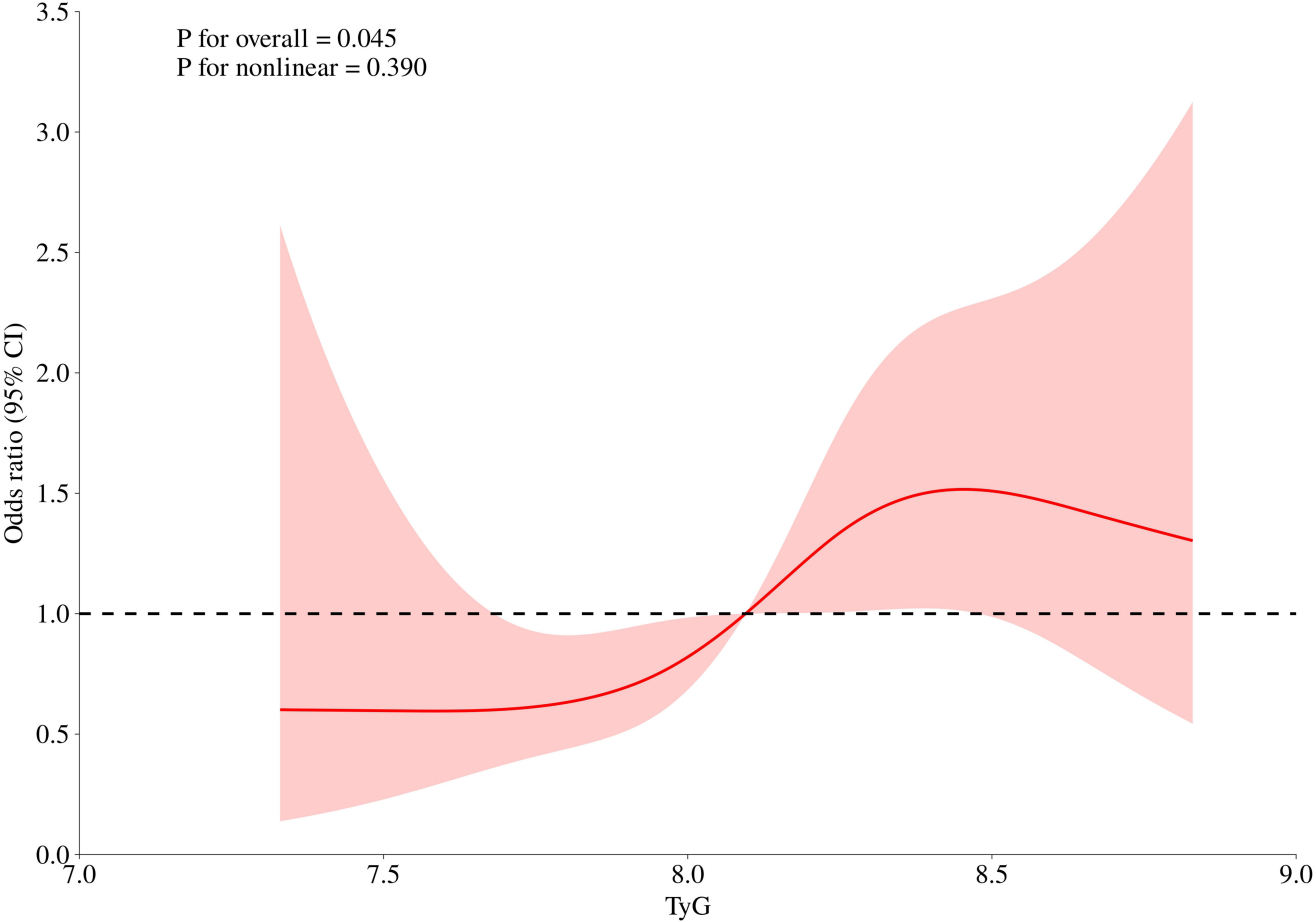
RCS plot of the association between the TyG index and the risk of HUA risk fully adjusted by age, sex, BMI, smoking, drinking, eGFR and LDL-C. The solid line represents the multivariable-adjusted odds ratio, the dashed line represents the reference line (OR = 1.0), and the shaded area indicates the 95% confidence intervals.

Furthermore, we employed RCS analysis to flexibly model and visualize the dose-response relationship between the TyG index and the risk of HUA (**Figure 4**). The RCS model indicated a marginally significant overall association (*P* for overall = 0.053). Importantly, the test for non-linearity was not statistically significant (*P* for non-linear = 0.217), confirming that the relationship between the TyG index and HUA risk is predominantly linear. This finding aligns consistently with the significant linear trend observed in our multivariable logistic regression models (*P* for trend = 0.041; continuous *P* = 0.026).

## 4 Discussion

In this study of 1,181 strictly metabolically healthy individuals—characterized by the complete absence of any individual MetS components (zero-component status)—we demonstrated a significant, positive association between the TyG index and the prevalence of HUA. Our findings reveal that the risk of HUA increases progressively with rising TyG levels, even after rigorous adjustment for traditional risk factors including BMI, eGFR, smoking status and drinking status.

### 4.1 Pathophysiological mechanisms in “healthy” individuals

The most striking finding of our study is that the TyG-HUA association persists in a population traditionally considered “metabolically healthy.” The TyG index serves as a robust surrogate for systemic and hepatic IR (20). The underlying pathological mechanism likely centers on the dual-pronged effect of insulin on renal and metabolic homeostasis.

From a renal perspective, hyperinsulinemia—secondary to IR—stimulates the urates transporter 1 (URAT1) and the sodium-dependent glucose cotransporter (SGLT) (21) in the proximal convoluted tubules. This insulin-mediated upregulation enhances the reabsorption of uric acid, thereby reducing fractional urate excretion and elevating serum levels (22). From a metabolic standpoint, the TyG index effectively captures the concurrent dysregulation of glucose and lipid metabolism. While traditional models link HUA primarily to overt obesity, our findings suggest that as the TyG index increases—even within an apparently healthy population—it reflects a progressive escalation toward systemic IR. Consistent with established metabolic pathways (23, 24), the lipid disturbances reflected by an elevated TyG index, specifically the oversupply of free fatty acids (FFAs), can impair the renal excretory capacity for uric acid. In parallel, the systemic IR represented by higher TyG levels further disrupts urate homeostasis by augmenting renal reabsorption (25, 26). Furthermore, even in individuals who do not meet clinical obesity criteria, a higher TyG index indicates a metabolic environment characterized by increased FFA flux and the secretion of pro-inflammatory cytokines, both of which act as potent drivers of hepatic *de novo* purine synthesis (27–29). Ultimately, the overarching state of IR, for which the TyG index is a sensitive proxy, serves as the central node linking these multi-faceted disturbances. By simultaneously accelerating hepatic urate production and hindering renal clearance, IR creates a “vicious cycle” of metabolic derangement. Our findings suggest that the risk of HUA increases in a linear manner as the TyG index rises, even within an apparently healthy population. This linear association underscores the importance of monitoring TyG levels across its entire range for early metabolic risk assessment.

### 4.2 Consistency and subgroup analyses

A key finding of this research is the consistent association between the TyG index and HUA risk across diverse clinical data. Our subgroup analyses demonstrated that the predictive value of the TyG index remained stable regardless of age, smoking status, or drinking status (*P* for interaction >0.05 for all). This consistency suggests that the TyG index reflects a fundamental metabolic derangement-early insulin resistance – that promotes urate elevation independently of common lifestyle confounders. Notably, although prior studies have suggested potential sex-related difference in uric acid metabolism (30)—our results showed no significant interaction between sex and the TyG index (*P* for interaction >0.05). This finding suggests that within this strictly metabolically healthy cohort, the TyG index serves as a consistent metabolic indicator for both men and women. We acknowledge that the association within the male subgroup did not reach statistical significance when analyzed independently. This is likely attributable to the limited sample size of male participants (14.28%) and the resulting lack of statistical power. However, the overall lack of interaction supports the universal potential of the TyG index as a tool for risk stratification across the entire apparently healthy population, rather than requiring sex-specific thresholds in this early subclinical stage.

The ROC analysis revealed that the TyG index exhibited fair discriminative ability for predicting HUA in our cohort, with an AUC of 0.631. While this performance may appear modest, its clinical significance is underscored by the high sensitivity (75.0%) and robust NPV (95.80%) observed at the optimal cut-off of 8.05. It is important to acknowledge that the low PPV (10.88%) indicates a high rate of false positives if the TyG index were used as a standalone diagnostic tool. However, this is mathematically expected given the relatively low prevalence of HUA (7.79%) in our strictly “metabolically clean” study population, where extreme metabolic derangements were absent.

The primary clinical utility of the TyG index in this context lies in its role as a potential tool for risk stratification in primary prevention settings, particularly for ruling out HUA. The high NPV allows clinicians to confidently exclude the risk of HUA in over 95% of healthy individuals scoring below the threshold. Even in the absence of traditional risk factors like hyperglycemia or hypertension, an elevated TyG index may identify individuals at a higher latent risk for HUA and subsequent cardiometabolic complications.

Our findings align with and extend the current understanding of SUA’s role in systemic health. While our study focuses on a “metabolically clean” cohort, the clinical relevance of monitoring SUA is emphasized by recent research in acute-care populations. For instance, Şengüldür et al. (3, 4) reported that SUA levels are closely linked to stroke risk and that even hypouricemia can serve as a predictor for 6-month mortality in emergency department patients. By demonstrating that the TyG index effectively identifies individuals at risk for HUA before the onset of traditional metabolic disorders, our study provides a potential preventive strategy to mitigate the long-term risks associated with SUA abnormalities observed in real-world clinical settings.

Therefore, while its specificity is limited—potentially due to the narrow metabolic range of our healthy cohort, the TyG index offers a simple, cost-effective method for early risk stratification in large-scale preventive screenings.

### 4.3 Limitations

Several limitations of this study must be acknowledged. First, the cross-sectional design precludes definitive conclusions regarding the temporal or causal relationship between the TyG index and HUA. Second, the sample size was relatively modest, and the study population was predominantly female (85.72%), which may limit the generalizability of our findings to the broader male population and potentially affect the statistical power of certain subgroup analyses. Third, the extensive exclusion of participants due to missing data resulted in a final cohort with a significantly healthier metabolic profile compared to those excluded. While this systematic difference may limit the generalizability of our findings to the general population, it allowed for a rigorous evaluation of the TyG-HUA association in a stringently defined “zero-component” metabolically healthy state. This suggests that the TyG index serves as a sensitive early biomarker even before the onset of clinically detectable metabolic derangements, providing a conservative yet robust estimate of the IR-urate relationship. Fourth, we cannot entirely rule out residual confounding from unmeasured factors such as dietary habits or specific physical activity levels. Finally, although the TyG index is a validated proxy for IR, we did not perform the hyperinsulinemic-euglycemic clamp, the gold standard for measuring IR. Furthermore, despite the cross-sectional nature of this study, our internal validation via bootstrap iterations demonstrated stable ROC performance; however, these performance estimates, including the specific AUC and cut-off value, should still be interpreted with caution and require further validation in prospective longitudinal cohorts and diverse ethnic groups.

### 4.4 Conclusion

In conclusion, the TyG index is independently and positively associated with the prevalence of HUA among apparently healthy individuals. The stability of this association across various subgroups reinforces the reliability of the TyG index as an early metabolic biomarker. Our findings highlight its potential as a tool for risk stratification in primary prevention, supporting the transition toward more robust and universal screening strategies for subclinical populations.

## Data Availability

The raw data supporting the conclusions of this article will be made available by the authors, without undue reservation.
